# Statins and Venous Thrombosis: A Story Too Good to Be True?

**DOI:** 10.1371/journal.pmed.1001311

**Published:** 2012-09-18

**Authors:** Frits R. Rosendaal

**Affiliations:** Department of Clinical Epidemiology and Department of Thrombosis and Haemostasis, Leiden University Medical Center, Leiden, The Netherlands

## Abstract

Frits Rosendaal comments on new findings from a meta-analysis conducted by Kazem Rahimi and colleagues, evaluating whether statins can prevent venous thromboembolism.

**Linked Research Article**

This Perspective discusses the following new study published in *PLOS Medicine*:

Rahimi K, Bhala N, Kamphuisen P, Emberson J, Biere-Rafi S, et al. (2012) Effect of Statins on Venous Thromboembolic Events: A Meta-analysis of Published and Unpublished Evidence from Randomised Controlled Trials. PLoS Med 9(9): e1001310. doi:10.1371/journal.pmed.1001310

A systematic review and meta-analysis conducted by Kazem Rahimi and colleagues re-evaluates the hypothesis, generated in previous studies, that statins may reduce the risk of venous thrombotic events. Their meta-analysis does not support the previous findings.

In this week's *PLOS Medicine* [1], Kazem Rahimi and colleagues report on a meta-analysis of randomised controlled trials to investigate the effect of statins on the occurrence of venous thrombosis. They included 29 trials in the analyses, for a total of nearly 150,000 randomised individuals. Overall, they found no or at most a small reduction in the incidence of venous thrombosis.

Venous thrombosis, which manifests mainly as deep vein thrombosis and pulmonary embolism, is a common, serious, and underestimated disorder. It occurs in one per 1,000 per year worldwide. Its main causes are related either to trauma and immobilisation, or to increased blood coagulability. In a substantial number of cases it occurs idiopathically, and it may be acutely fatal [2]. It has severe long-term consequences: one study estimated that after one year, 12%–20% of patients with venous thrombosis had died [3]. Up to half of patients with deep vein thrombosis of the leg develop post-thrombotic syndrome, with symptoms that vary from pain to ulceration. And, finally, around 5% of patients each year experience a repeat thrombosis [4],[5]. In 2008, the United States Surgeon General issued a “call to action” to prevent deep vein thrombosis and pulmonary embolism, and concluded that awareness of these treatable disorders was too low [6]. Indeed, prevention and treatment of venous thrombosis is straightforward: use of anticoagulants will reduce the risk to nearly zero. However, anticoagulants also cause bleeding, and are among the top 10 drugs causing serious side effects [7]. So far, all anticoagulants follow Åstrup's dogma of the haemostatic balance of the coagulation system between bleeding and clotting [8], i.e., any factor that tilts the balance towards an antithrombotic effect will also cause bleeding. This severely limits the long-term use of anticoagulant drugs. A drug with antithrombotic properties that does not increase the risk of bleeding would offer tremendous therapeutic opportunities, but sounds too good to be true.

Are statins like that? Several observational studies have shown that statin users have lower risk of venous thrombosis than non-users [9]–[11]. This was confirmed in a secondary analysis of a randomised trial of statins versus placebo [12]. There are no known effects of statins on the coagulation system, nor does it seem that statin users have an increased risk of bleeding. With a risk reduction of 50% as observed in these observational studies, statins seem to offer an attractive option for long-term prevention of venous thrombosis in patients with an intermediate risk.

Stories too good to be true may nevertheless be true, but need to be approached with ample skepticism. Over the years, beneficial effects have been attributed to statins that go beyond lipid-lowering. These include heart failure, arrhythmia, multiple sclerosis, depression, Alzheimer's dementia, osteoporosis, osteoarthritis, macular degeneration, sepsis, infections, acute lung injury, neuropathic pain, AIDS, fatty liver disease, and epilepsy. To paraphrase Anton Chekhow, who said that when many remedies are used to treat a disease, it means the disease is incurable, one may argue that if many cures are attributed to a single drug, it may be ineffective—and non-causal explanations should be sought. A “healthy user effect” has been proposed to explain this unlikely pleiotropic action, i.e., that statins are prescribed preferentially to individuals with a favourable risk profile [13], or that the healthiest users are analysed in some observational studies [14]. Although users of a drug are rarely more healthy than non-users, a widespread primary prevention use may introduce such bias. When this type of confounding by indication is suspected, a randomised trial is the accepted method to break the link between drug prescription and prognosis. A secondary analysis of the JUPITER trial indeed showed a risk reduction of venous thrombosis with statin use, of about 40% [12].

In the absence of any randomised trials with venous thrombosis as the primary endpoint, Rahimi and colleagues present a pooled analysis of 29 randomised studies, all with primary endpoints other than venous thrombosis. Only in two studies had venous thrombotic events been presented in the published report, but they had been recorded as adverse events in all other trials, too, and these data were made available to the authors. When comparing statin users with non-users in the meta-analysis, venous thrombosis occurred in 0.9 per 1,000 in statin users versus 1.0 per 1,000 in non-users, for an odds ratio of 0.89 (95% confidence interval 0.78–1.01). When the study prompting this analysis (JUPITER, [12]) was excluded, the odds ratio became 0.93 (95% CI, 0.82–1.07). Whereas these results perhaps still suggest a small effect, no effect whatsoever was observed in trials comparing high versus low dose statins.

Side effects of drugs are not necessarily class effects, particularly when the mechanism of the side effect differs from the primary mechanism of the drug, so possibly only some statins reduce thrombotic risk. Therefore, an analysis was done by type of statin, which suggested a difference between rosuvastatin and the other statins, with an OR for rosuvastatin of 0.60 (95% CI 0.39–0.92) for all trials of statins versus no statins.

The analysis by Rahimi and colleagues is completely based on data from randomised studies and is therefore more credible than observational evidence since it excludes confounding by indication. Usually, side effects of drugs are unintended and unexpected, and therefore prescription is not related to prognosis, in which case observational analyses will yield valid results, because users and non-users are comparable on relevant characteristics (no confounding). However, the wide range of positive effects reported for statins strongly suggest incomparability of users and non-users, for instance introduced by a healthy user effect, in which case a seemingly positive effect of the drug is the result of the better risk profile of users. Nevertheless, while this confounding is solved by the use of randomised data, the analysis of Rahimi and colleagues may also suffer from bias due to outcome misclassification. It is possible that some venous thrombotic events go undetected in trials that do not have venous thrombosis as the focus of interest. In double-blind trials such misclassification would be random, but even then it would mask an effect by introducing noise, resulting in an underestimation of the effect. If this has happened, the thrombosis reducing effect of statins may be larger than the 11% reported here.

Even if this study does not provide a definite answer, what can we tentatively conclude? Firstly, that for the association between statins and venous thrombosis the methodologically strongest analysis shows at most a very small effect. Secondly, if we do not wish to discard the possibility of a beneficial effect for the whole class, any such effects are limited to rosuvastatin.

In sum, the dogma of the haemostatic balance still holds: effective anticoagulants cause bleeding. However, for those who like good stories of safe antithrombotic drugs, there is still some room to hope that the story may eventually not be too good to be true.
